# Lymphotoxin-sensitive microenvironments in homeostasis and inflammation

**DOI:** 10.3389/fimmu.2012.00243

**Published:** 2012-07-31

**Authors:** Bryant Boulianne, Elisa A. Porfilio, Natalia Pikor, Jennifer L. Gommerman

**Affiliations:** Department of Immunology, University of Toronto,Toronto, ON, Canada

**Keywords:** lymphotoxin, follicular dendritic cell, fibroblastic reticular cell, lymph node, chemokine, follicle-like structures

## Abstract

Stromal cell microenvironments within lymphoid tissues are designed to support immune cell homeostasis and to regulate ongoing immune responses to pathogens. Such stromal cell networks have been best characterized within lymphoid tissues including the spleen and peripheral lymph nodes, and systems for classifying stromal cell phenotypes and functions are emerging. In response to inflammation, stromal cell networks within lymphoid tissues change in order to accommodate and regulate lymphocyte activation. Local inflammation in non-lymphoid tissues can also induce *de novo* formation of lymphoid aggregates, which we term here “follicle-like structures.” Of note, the stromal cell networks that underpin such follicles are not as well characterized and may be different depending on the anatomical site. However, one common element that is integral to the maintenance of stromal cell environments, either in lymphoid tissue or in extra-lymphoid sites, is the constitutive regulation of stromal cell phenotype and/or function by the lymphotoxin (LT) pathway. Here we discuss how the LT pathway influences stromal cell environments both in homeostasis and in the context of inflammation in lymphoid and non-lymphoid tissues.

## INTRODUCTION

Within the secondary lymphoid tissues, stromal cell networks are an integral scaffold for complex immune cell interactions necessary to mount an effective immune response to pathogens. The maintenance of the phenotype and function of some stromal cell types is critically dependent on constitutive signaling of the lymphotoxin-beta receptor (LTβR). LTβR is a member of the tumor necrosis factor (TNF) superfamily of receptors and is triggered by two ligands: membrane-bound LTα_1_β_2_ heterotrimers and LIGHT, resulting in the activation of both the canonical and alternative NFκB pathways (Bista et al., 2010). During embryogenesis, the LTβR-dependent activation of NFκB within lymphoid tissue organizer (LTo) cells is achieved by interaction with LTαβ-expressing lymphoid tissue inducer (LTi) cells, thus facilitating lymph node (LN) and Peyer’s patched (PP) development (Mebius, 2003; Ruddle and Akirav, 2009).

In the adult animal, stromal cell phenotype and function must be constitutively maintained for the lifetime of the host in order to maintain the integrity of lymphoid tissue, and much of this maintenance is accomplished by continual LTβR signaling (Gommerman and Browning, 2003). The cell types which provide LTαβ are generally lymphocytes, in particular B cells (Tumanov et al., 2002, 2004), but can also be LTi-like innate lymphoid cells, especially in the context of the gut (Eberl, 2005). The moment such a homeostatic program is interrupted, as achieved by a single injection of the LT pathway antagonist LTβR-Ig, stromal cell networks collapse and the lymphoid tissues become disorganized (Mackay and Browning, 1998). When the drug is cleared, however, aspects of the lymphoid tissue stromal cell environment can be re-established (Gommerman et al., 2002).

These findings have important implications for how we view stromal cells. First, it suggests that stromal cells are highly dynamic and rely on continual input from LTαβ-expressing cells. Second, since LTαβ is up-regulated on activated lymphocytes (Summers-DeLuca et al., 2007), lymphocytes that have been triggered by foreign or self-antigen (Ag) may have the potential to provide stromal cell differentiation cues. Finally, the ability to manipulate stromal cell biology via the LT pathway allows one to study the potential function of LT-sensitive stromal cell types during tissue homeostasis and during inflammation. Here, we outline the role of LTβR signaling in the homeostatic maintenance of non-lymphoid cell types within LN and in the small intestine, and explore how LTβR signaling influences changes in stromal cell phenotype/function during inflammation within lymphoid tissues and in ectopic sites of follicle development.

## LTβR-DEPENDENT REGULATION OF STROMAL CELLS IN PERIPHERAL LYMPHOID TISSUES

**FIGURE 1:**
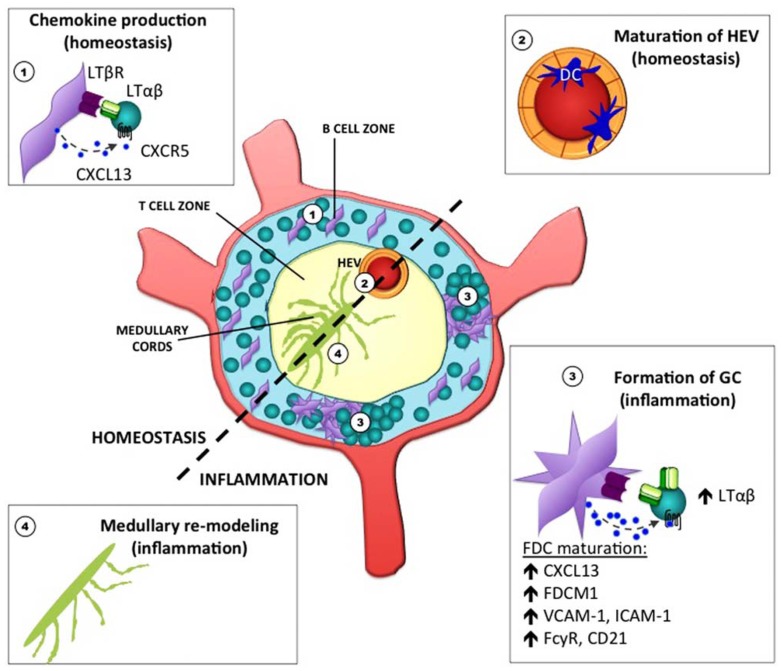
**Stromal cell elements in the lymph node under lymphotoxin control during homeostasis and inflammation.** The LT pathway is critical for the proper maintenance and function of various stromal cell elements in the LN. During homeostasis, chemokine production by FDC in the primary follicle is required for B cell positioning (1). LTβR signaling in endothelial cells of HEV is also required for the expression of sulfotransferases that promote the proper glycosylation of PNAd (2). During inflammation, the LN becomes enlarged, stromal cells acquire new functions, and increased vascularization occurs (not depicted). In addition, clusters of B and T cells aggregate within germinal centers during T-dependent immune responses, and highly differentiated FDC within the GC environment require LTβR signaling (3). To facilitate the output of plasma cells that emerge from these GC reactions, remodeling of the medullary region has been shown to occur (4).

Lymph nodes are composed of a variety of stromal cell types whose phenotype and function are being increasingly elucidated (Malhotra et al., 2012). In general, marginal reticular cells are located in the sub-capsular sinus (SCS), under which follicular dendritic cells (FDCs) populate the follicle. Fibroblastic reticular cells (FRCs) are located in the T cell-rich paracortex area and LN medullary fibroblasts are found in the medullary cords. Vascular and lymphatic endothelial cells are an additional source of non-lymphoid cell types. In the context of the non-inflamed LN, we focus on FDCs, FRCs, and the endothelial cells that form high endothelial venules (HEV) since the role of the LT pathway in these cell types has been well described. Depicted in **Figure 1** are examples of LN stroma that are under LT control in both the steady state and during inflammation.

### FOLLICULAR DENDRITIC CELLS

B cell follicles in lymphoid tissues are largely defined by FDC (Allen and Cyster, 2008). FDCs are an important source of the B cell chemo-attractant CXCL13 which helps to establish the polarity between B and T cell zones in lymphoid tissues. FDCs also aid in germinal center responses by secreting the B cell survival factor BAFF and by trapping immune complexes for display to activated B cells (Suzuki et al., 2010). Though the exact identity of the FDC precursor is still unclear, it is thought that FDCs derive from mesenchymal cells *in situ *(Munoz-Fernandez et al., 2006; Allen and Cyster, 2008). It is well established that mature primary FDCs are maintained within B cell follicles by virtue of the interaction between LTαβ on B cells and LTβR on a resident radio-resistant stromal cell precursor (Fu et al., 1998; Gonzalez et al., 1998; Endres et al., 1999). LTβR signals stimulate FDCs to secrete CXCL13, which attracts more B cells and induces them to up-regulate LTαβ, thereby initiating a positive feedback loop (Ansel et al., 2000). Constitutive signaling is required for FDC maintenance and disruption of LTαβ-LTβR signaling *in vivo *results in the rapid disappearance of FDCs along with a disorganization of the B cell and T cell zones (Mackay et al., 1997).

### FIBROBLASTIC RETICULAR CELLS

Fibroblastic reticular cells are found predominantly in the T cell areas of LN (Balogh et al., 2008; Turley et al., 2010). FRCs secrete fibronectin, laminin, and ER-TR7 antigen, which bind ECM collagen fibers to produce a reticular network (Katakai et al., 2004). This reticular network serves as a scaffold for cell migration and retention (Bajenoff et al., 2006), provides a source of IL-7 (Link et al., 2007), creates conduits that facilitate movement of chemokines and small soluble Ag (Roozendaal et al., 2009), and influences T cell tolerance in the steady state (Fletcher et al., 2011). Like FDCs, FRCs are thought to derive *in situ *from a mesenchymal precursor, and multipotent mesenchymal stem cells isolated from human tonsils and bone marrow stimulated with recombinant TNFα and LTαβ develop an FRC phenotype *in vitro *(Ame-Thomas et al., 2007). Murine FRCs cultured alone *in vitro* do not secrete ER-TR7 but upon co-culture with CD4^+^ T cells FRCs produce large amounts of reticula that are coated with ER-TR7 in an LT- and TNFα-dependent manner (Katakai et al., 2004). Similarly, LTβR-Ig treatment diminished FRC networks in pancreatic infiltrates of diabetic CXCL13-RIP mice* in vivo *(Link et al., 2011). However, it is unclear if the development and/or maintenance of an intact ER-TR7-producing FRC network within LN requires constitutive LTβR signaling, although the loss of T cells concomitant with a decrease in LTαβ is correlated with FRC collapse in human immunodeficiency virus (HIV) infection (Zeng et al., 2012).

### HIGH ENDOTHELIAL VENULES

High endothelial venules are the portals of entry for naive lymphocytes into LN. This is because the endothelium of HEV displays adhesion molecules, notably peripheral node addressin (PNAd). Mice that receive LTβR-Ig treatment have hypo-cellular LN due to the requirement of LTβR signaling in regulating the expression of sulfotransferase enzymes that mediate post-translational modification of PNAd. Without these modifications, PNAd is aberrantly expressed in HEV and naive L-selectin^+^ lymphocytes transmigrate into LN tissues inefficiently (Browning et al., 2005). A similar paradigm is observed for ectopic lymphoid aggregates in the pancreas (Drayton et al., 2003). Recently, it was shown that dendritic cells (DC) are an important source of LTαβ in providing the maturation signal for HEV. This suggests there could be intimate cross-talk between DC and HEV (Moussion and Girard, 2011). Whether DC can communicate with other LTβR-expressing stromal cell elements within lymphoid tissues remains to be determined.

## LTβR-DEPENDENT REGULATION OF STROMAL CELLS IN THE SMALL INTESTINE

The LT pathway plays a critical role in regulation of IgA production in the gut (Kang et al., 2002), and this has been linked to the activity of LTβR signaling in gut-resident stromal cells in different types of gut-associated lymphoid tissues (Tsuji et al., 2008). Such lymphoid tissues include PP, which are located along the small intestine. PP contains large B cell follicles along with smaller T cell regions in “inter-follicular” zones. Not unlike the case in LN, FDC and T/B segregation within the PP are likewise dependent on LTβR signaling in PP stromal cells, primarily by virtue of expression of LTαβ on B cells (Tumanov et al., 2004). PP-resident FDCs are somewhat different than LN FDCs in that they produce mediators that particularly encourage IgA class switch recombination (Suzuki et al., 2010). Overarching the PP follicles is the sub-epithelial dome that hosts a rich community of DC. Interestingly, expression of the chemokine CCL20 in the follicle-associated epithelium which overlies the DC-rich sub-epithelial dome is also LT sensitive (Rumbo et al., 2004). The CCL20/CCR6 axis may be important for the recruitment of B cells to the PP, and since B cells can express LTαβ, this could potentially drive the subsequent organization of the PP architecture (Williams, 2006). Microfold (M) cells, which are also partially dependent on the LT pathway (Debard et al., 2001), are interspersed within the follicle-associated epithelium. Along with dome-resident DC, M cells play an important role in shuttling Ag from the gut lumen into the PP for sampling and generation of immune responses. In general, the stroma in PP is less well characterized than in the LN.

Also within the small intestine are lymphoid tissue structures that develop strictly after birth called cryptopatches. In the presence of commensal bacteria, these cryptopatches mature to become isolated lymphoid follicles (ILF; Taylor and Williams, 2005). LTαβ- and LTβR-deficient animals lack both ILF and cryptopatches. It is thought that IL-7 release by the underlying stroma in the small intestinal lamina propria induces the expression of LTαβ on LTi-like innate lymphoid cells. This in turn results in the triggering of LTβR to form the cryptopatch which matures into an ILF (Eberl, 2005). Like PP, ILF development also requires the CCL20/CCR6 axis (Bouskra et al., 2008). Such ILF can be an alternative location for the generation of mucosal IgA^+^ cells (Tsuji et al., 2008).

## LTβR-DEPENDENT CHANGES IN LYMPHOID STROMAL CELLS DURING INFECTION AND INFLAMMATION

Several changes occur in the draining inflamed LN following exposure to Ag in adjuvant: systems for Ag transport are mobilized, stromal cells acquire new functions, the LN becomes enlarged, neo-vascularization occurs to accommodate increased cellular input, and specialized niches that support T/B interactions are formed. In this section we describe these changes, how such changes are influenced by different types of stromal cells, and the role of the LT pathway in orchestrating dynamic changes in the inflamed LN.

### ANTIGEN TRANSPORT

Lymph-borne Ag enters LN into the SCS. There, Ag complexes are bound by CD169^+^F4/80^-^ SCS macrophages (SCS Mφ) that extend their processes into the SCS lumen to pick up Ag complexes (Carrasco and Batista, 2007; Junt et al., 2007). Non-cognate B cells subsequently pick up Ag complexes from SCS Mφ, carry them deeper into follicles, and deposit the Ag on FDCs in germinal centers (Phan et al., 2007). Interruption of this transport chain results in early dissipation of germinal centers and impaired affinity maturation. SCS Mφ express LTβR and their presence in the SCS region requires signals from LTαβ on B cells (Phan et al., 2009). As such, the expression of LTαβ on B cells is an important form of innate defense due to its ability to signal LTβR on cells within the SCS: the first point of Ag entry (Moseman et al., 2012). Stromal cells within the SCS have been described (Katakai et al., 2008), and it will be of interest to learn how these stromal cells interact with the Ag transport chain.

### LYMPHOID TISSUE REMODELING DURING INFLAMMATION AND INFECTION

Dramatic changes occur in lymphoid tissues in response to viral infections. For example, during lymphocytic choriomeningitis virus (LCMV) infection, lymphoid tissue architecture becomes disorganized but is eventually restored in a manner that depends on LTαβ expression on LTi-like innate lymphoid cells (Scandella et al., 2008). In addition to this dramatic remodeling, lymphoid stroma can be an important source of type I interferons during viral infection, and LTβR signaling in splenic stroma can drive such a Type I interferon response independent of MyD88 or TRIF-derived signals (Schneider et al., 2008).

In the LN, inflammation also greatly increases the size of the LN and this LN hypertrophy is accompanied by endothelial cell proliferation that can be promoted by the production of VEGF. FRC is a source of VEGF and this is dependent on LTαβ/LTβR signaling (Chyou et al., 2008) as well as input by the alternative LTβR ligand LIGHT (Zhu et al., 2011). Furthermore, LTαβ expression on B cells can also drive HEV network extension/remodeling in response to LCMV infection independent of VEGF (Kumar et al., 2010). Thus, through various mechanisms, the LT pathway is important for accommodating the increased flow of lymphocytes into a draining reactive LN. The medullary stroma, which supports lymphocyte egress from the LN, also becomes remodeled during an immune response. This may be important for providing a niche for the incredible burst in plasma cell output that is generated following a germinal center response. In this process, collagen-poor and collagen-rich areas are created, with plasma cells settling in the collagen-rich regions, presumably to take advantage of stromal cell factors that may enhance their survival (Zhu et al., 2011).

### GERMINAL CENTER FORMATION

As mentioned, mature primary FDCs are located throughout B cell follicles and rely on constitutive, low-level LTβR signaling (Fu et al., 1998; Gonzalez et al., 1998; Endres et al., 1999). During an immune response, activated Ag-specific B cells that receive co-stimulation from T cells up-regulate LTαβ even further and provide stronger LTβR signals to FDCs (Vu et al., 2008). This elevated LTβR signaling prompts FDCs to mature into secondary FDCs within germinal centers. Secondary FDCs up-regulate complement receptors CD21 and CD35 as well as FcγRIIB to enhance capture of Ag complexes (Allen and Cyster, 2008). While the exact role for Ag complexes on FDCs is still debated, it is likely that they help sustain the germinal center response and enhance affinity maturation. Secondary FDCs also begin to express FDC-M1 antigen (Mfg-e8), which may play a role in the clearance of apoptotic germinal center B cells (Kranich et al., 2008).

## INFLUENCE OF LTβR SIGNALING ON ECTOPIC LYMPHOID TISSUE

Inflammation in peripheral tissues can create an environment that is permissive to the formation of follicle-like structures (FLS). These structures have been observed in a wide variety of settings and display differing levels of organization, and in some cases have been shown to support local immune responses (Aloisi and Pujol-Borrell, 2006). In this section, we review two examples of FLS and speculate on how the LT pathway may support such structures.

### INDUCIBLE BRONCHIAL LYMPHOID TISSUE

Inducible bronchus-associated lymphoid tissues (iBALT; Randall, 2010) are FLS that form in the lungs in response to respiratory inflammation due to infection (Moyron-Quiroz et al., 2004; Lugade et al., 2011), chronic inflammation (Hogg et al., 2004), or autoimmunity (Rangel-Moreno et al., 2006). The content of such structures varies from highly organized niches beneath a dome epithelium with defined T cell and B cell areas and FDC capable of supporting germinal centers, to small clusters of lymphocytes containing mostly B cells and some FDC (Moyron-Quiroz et al., 2004). Local production of CXCL13, CCL19, and CCL21 drives the recruitment of lymphocytes to iBALT follicles (Foo and Phipps, 2010). Fully formed iBALT require approximately 10 days to become organized niches in adult mice post-infection (Moyron-Quiroz et al., 2004; Halle et al., 2009) but are maintained for months (Moyron-Quiroz et al., 2006).

Unlike LN and PP which require LTαβ–LTβR signaling for their formation, studies using *LTα*^-^^/^^-^ mice have shown that LTβR signaling is not required for iBALT formation or induction of CXCL13, CCL19, and CCL21 during acute inflammation (Moyron-Quiroz et al., 2004). Instead, Randall and colleagues determined that CD4^+^IL-17^+^ cells are necessary to initiate iBALT formation (Moyron-Quiroz et al., 2004). However, once established, CD4^+^IL-17^+^ cells are insufficient for optimal organization and maintenance of iBALT which instead is dependent on LTβR signaling.

### FLS IN THE CENTRAL NERVOUS SYSTEM

Follicle-like structures have been documented at sites of chronic inflammation in several autoimmune diseases including: rheumatoid arthritis, Sjörgen’s syndrome, systemic lupus erythematosus, and Multiple Sclerosis (MS; Aloisi and Pujol-Borrell, 2006). There is a range in the level of lymphoid-like organization of these structures: from perivascular infiltrates, to diffuse aggregates with HEV-like vessels, to organized follicles with T and B cell segregation and underlying FDC networks (Browning, 2008). The disease relevance of FLS is associated with local tissue injury and cell death. In MS, FLS preferentially accumulate in the meninges in patients at the later progressive stage of the disease (Serafini et al., 2004), and meningeal FLS are associated with increased demyelination and neuronal loss (Magliozzi et al., 2007, 2010).

A role for the LT pathway in attenuating clinical disease has previously been described in the rodent model of MS, experimental autoimmune encephalomyelitis (EAE; Gommerman et al., 2003). Pharmacological disruption of LT signaling reduces the size and number of meningeal FLS compared with control treatment (Columba-Cabezas et al., 2006). Impaired FLS formation following LT inhibition is concomitant with reduced mRNA levels of CXCL10 and CXCL13 in the brain, suggesting that LT regulates chemokine induction at peripheral sights of inflammation. However, not unlike iBALT, emerging studies in EAE also support the notion that distinct pathways may culminate in orchestrating FLS. For example, adoptively transferred myelin-specific Th17 cells induce EAE concomitant with FLS formation (Peters et al., 2011). How signals from the LT pathway and from Th17 cells co-integrate to induce and/or maintain FLS structures in the CNS is unknown.

## CONCLUSION

It is clear that LTβR-generated signaling underpins the maintenance and in some cases the function of stromal cell types within lymphoid tissues. Not discussed here are examples of how LTβR signaling is also important in myeloid/DC biology (Deluca and Gommerman, 2012), and DC have been implicated in regulating stromal cells and the formation of FLS (GeurtsvanKessel et al., 2009; Halle et al., 2009; Moussion and Girard, 2011). Thus, it will be of interest to learn more about the connections between DC and stromal cells with respect to the LT pathway. Moreover, many questions remain unanswered regarding how the LT pathway integrates with other forms of input, such as Th17 cells, to orchestrate distinct stages of FLS formation (i.e., initiation versus maintenance), and which LTαβ and LTβR-expressing cell types support FLS. Indeed, while exciting advances have been made toward understanding the nature of stromal cell types in peripheral LN, this question has barely been addressed in the mucosal lymphoid tissues and in the context of FLS. Unraveling the many facets of LTβR signaling in regulating and fine-tuning the immune response is a tall order, but of value for considering the therapeutic potential of LT inhibitors in treatment of chronic diseases.

## Conflict of Interest Statement

The authors declare that the research was conducted in the absence of any commercial or financial relationships that could be construed as a potential conflict of interest.
